# An Innovative Retrieval-Augmented Generation Framework for Stage-Specific Knowledge Translation in Biomimicry Design

**DOI:** 10.3390/biomimetics10090626

**Published:** 2025-09-17

**Authors:** Hsueh-Kuan Chen, Hung-Hsiang Wang

**Affiliations:** Department of Industrial Design, National Taipei University of Technology, Taipei 10608, Taiwan; t112588026@ntut.org.tw

**Keywords:** artificial intelligence in design, biomimicry, biomimetics, biologically-inspired design, interdisciplinarity, automotive design, retrieval-augmented generation, large language models

## Abstract

Converting biological strategies into practical design principles during the Discover–Abstract phase of the Biomimicry Design Spiral (BSD) presents a considerable obstacle, particularly for designers lacking a biological background. This research introduces a Retrieval-Augmented Generation (RAG) framework that combines a specialized AskNature database of 2106 documents with a locally executed Llama 3.1 large language model (LLM) to fill this void. The innovation of this study lies in integrating the BDS with a stage-specific RAG–LLM framework. Unlike BioTRIZ or SAPPhIRE, which require specialized expertise, our approach provides designers with semantically precise and biologically grounded strategies that can be directly translated into practical design principles. A quasi-experimental study with 30 industrial design students assessed three setups—LLM-only, RAG-Small, and RAG-Large—throughout six biomimicry design stages. Performance was assessed via expert evaluations of text and design concept quality, along with a review of retrieval diversity. Findings indicate that RAG-Large consistently yielded superior text quality in stages with high cognitive demands. It also retrieved a more varied array of high-specificity biological ideas and facilitated more coherent incorporation of functional, aesthetic, and semantic aspects in design results. This framework diminishes cognitive burden, boosts the relevance and originality of inspirations, and provides a reproducible, stage-specific AI assistance model for closing the knowledge translation gap in biomimicry design, though its current validation is limited to a small sample and a single task domain.

## 1. Introduction

Design innovation is undergoing a profound transformation in the age of artificial intelligence (AI) and data-centric development. Recent advances in large language models (LLMs) and generative design methodologies are reshaping how designers explore ideas and translate knowledge into practical product solutions [1,2]. While sectors like the automotive industry are increasingly adopting data-focused processes to accelerate development timelines and adapt to changing market demands, small and medium-sized enterprises (SMEs) and independent designers often stick to traditional, precedent-based methods. These conventional approaches lack the speed, scalability, and comprehensive knowledge integration essential for innovation in knowledge-intensive fields. Biomimicry—which draws on natural structures, functions, and behaviors—provides unique opportunities for improving energy efficiency, structural optimization, and creating distinctive product semantics [3].

However, the challenge of converting biological insights into applicable design principles remains a significant obstacle. The Discover–Abstract stage of the Biomimicry Design Spiral (BDS) involves identifying and interpreting biological strategies before applying them in engineering contexts. There have been numerous reports of incorrect analogical transfers, where non-biologists mistakenly align functionally irrelevant biological processes with design issues [4]. Though platforms like AskNature curate biological strategies, their keyword-centric search functions and fragmented knowledge frameworks may hamper efficiency, especially for SMEs and designers lacking specialized biological knowledge. Retrieval-Augmented Generation (RAG) presents an opportune solution to these issues. By merging semantic retrieval with generative reasoning, RAG mitigates the occurrence of hallucinations in LLM outputs and enhances the precision of knowledge-intensive tasks [2,5]. Within design settings, RAG can bridge the divide between extensive biomimetic databases and creative concept formation, providing better access, filtering, and translation of biological strategies. Additional techniques, such as text-to-image (T2I) generation, facilitate visualization and iteration during initial design phases, enhancing communication and concept evaluation.

Despite these advancements, the incorporation of RAG into biomimetic design processes—particularly during the Discover and Abstract phases—remains scarcely examined. Few studies have explored how RAG-enhanced retrieval impacts designers’ ability to translate knowledge or its effect on the visual and functional precision of biomimetic results. To more clearly position the innovation of this study, we emphasize that prior biomimicry frameworks—such as BioTRIZ and SAPPhIRE/BIDARA—offer systematic reasoning but remain too complex for widespread adoption by industrial designers without engineering or biological expertise. Recent attempts to apply RAG in design (e.g., Majumder et al. [6]; Toukmaji & Tee [7]) have shown potential for analogical reasoning and conceptual modeling, yet they operate as generic retrieval pipelines without direct stage alignment to validated design methodologies. In contrast, our framework is the first to embed RAG within the BDS in a stage-dependent manner, ensuring that retrieval and generation are contextually adapted to each phase. By contrast, our contribution is to integrate the widely used BDS with a RAG–LLM framework, specifically designed to reduce the Discover–Abstract bottleneck. Unlike existing keyword-based retrieval from AskNature, our stage-specific RAG method provides semantically precise, context-rich biological strategies and translates them into practical design principles. This combination of BDS with RAG-LLM represents a novel, reproducible pathway that supports designers in generating biologically coherent and innovative concepts, while lowering the expertise barrier for non-biologists.

To fill this void, this research develops an integrated RAG–LLM pipeline specifically for biomimetic automotive design. We assess how the scale of retrieval influences translation efficiency and the quality of concepts, using AskNature’s Biological Strategies and Innovations repositories as the knowledge foundation. We also integrate T2I generation and 2D-to-3D transformation to test whether RAG-augmented text outputs can generate more innovative and biologically coherent design proposals. By aligning the Double Diamond model (DDM) with the BDS, this work proposes a structured, data-driven framework to enhance AI-assisted biomimicry and speed up design innovation.

## 2. Related Work

### 2.1. Biomimicry Design Model, Databases, and Applications

Biomimicry offers a structured approach to converting biological insights into pioneering and sustainable engineering solutions. Among the available frameworks, the BDS, crafted by the Biomimicry Institute, stands out as the most widely utilized due to its clear six-step sequence—Define, Biologize, Discover, Abstract, Emulate, and Evaluate [3,8]. This iterative approach fosters both expansive exploration and focused refinement, making it suitable for diverse design challenges. Nonetheless, the transition from Discover to Abstract is particularly challenging, requiring designers to derive functional principles from biological information, necessitating considerable biological expertise [4]. Other methods provide more systematic reasoning. BioTRIZ, derived from the TRIZ theory of inventive problem-solving, promotes functional enhancement by aligning biological strategies with engineering conflicts [9,10]. SAPPhIRE/BIDARA utilizes a causal modeling framework—System, Action, Part, Phenomenon, Input, oRgan, and Effect—to depict biological processes, facilitating computational reasoning and database-based knowledge extraction [7,11,12]. While effective for engineering purposes, their intricacy poses challenges for adoption by designers and small-to-medium enterprises (SMEs).

Table 1 presents a comparison of these models, detailing their structures, engineering relevance, and major drawbacks. BDS is broadly accepted and effective for multidisciplinary teams but depends heavily on manual biological knowledge retrieval and abstraction. BioTRIZ and SAPPhIRE offer systematic or computational reasoning but necessitate specialized skills. Considering these aspects, the study adopts BDS as the primary framework given its widespread use in design practice, with a focus on addressing its main shortcoming—inefficient knowledge retrieval in the Discover–Abstract stage—through AI-supported tools. Compared with BioTRIZ and SAPPhIRE/BIDARA, which provide structured reasoning but require significant expertise in engineering or biology, the proposed RAG–LLM framework is designed for accessibility by industrial designers. Its stage-specific integration with the BDS reduces complexity by delivering semantically relevant biological strategies directly within the design workflow. This approach lowers the barrier to applying biomimicry for small and medium-sized enterprises (SMEs) and non-biologist designers, thereby extending the practical reach of biomimetic methods.

The bottleneck in translating knowledge is significantly linked to the constraints of current biomimicry databases. The most prevalent platform, AskNature (https://AskNature.org, accessed on 1 August 2024), categorizes biological knowledge into Biological Strategies and Innovations, making it more accessible to non-experts in biology [3]. Nevertheless, its reliance on keyword searches and disjointed knowledge structure limits in-depth exploration and comprehensive reasoning. Designers are left to manually navigate through large amounts of loosely connected data, which is inefficient for small and medium enterprises (SMEs) and independent designers who need rapid, knowledge-driven innovation. Though challenges exist, biomimicry has shown significant engineering promise, notably in the automotive industry. A case in point is the Mercedes-Benz Bionic Car, which took inspiration from the boxfish (Ostraciidae). By emulating the boxfish’s hexagonal exoskeleton, the car achieved a low drag coefficient (Cd = 0.19) and improved structural strength while remaining lightweight [14]. However, follow-up studies offered more nuanced insights: Van Wassenbergh et al. [15] suggested that the boxfish’s body shape improves maneuverability through destabilization effects, while Chowdhury et al. [16] validated its drag-reduction benefits in vehicle applications. These findings highlight that biomimetic methods require contextual interpretation and empirical verification, emphasizing the necessity for tools that enhance the precision of knowledge translation at the initial design phases.

### 2.2. RAG for Biomimicry Knowledge Translation and Conceptual Design

The combination of RAG and LLMs delivers a scalable and intellect-enhancing approach to tackle the Discover–Abstract bottleneck faced by BDS. RAG draws semantically pertinent information from domain-specific sources, such as AskNature, and aids LLMs in creating design concepts that are informed by biology [2]. Researchers have crafted RAG workflows catered to specific domains. Toukmaji and Tee [7] introduced a workflow that corresponds with BDS stages, fostering analogical reasoning by simulating interactions between engineers and biologists using agent-based dialogue. Their findings indicated improvements in plausibility, functional reasoning, and interdisciplinary harmony in resulting design concepts. For automated abstraction, Majumder et al. [6] utilized RAG to produce SAPPhIRE models, connecting physical effects, functions, and behaviors from biological and engineering texts, illustrating RAG’s ability to support both conceptual modeling and functional reasoning in design. Nonetheless, Barnett et al. [17] pinpointed seven frequent failure points in RAG processes, such as hallucinations despite relevant data and prompt overload, affecting reliability—particularly in fields needing precision, like biomimicry. Agrawal et al. [18] tackled this with Mindful-RAG, an error-aware framework that diagnoses mistakes throughout input–retrieval–generation processes, enhancing interpretability and trust in RAG systems for bio-inspired design.

Evaluation techniques are advancing as well. Salemi and Zamani [19] presented eRAG, a document-level retrieval metric more closely related to generation quality than traditional top-k relevance scores, highlighting the critical role of relevance in accurate abstraction. RAG-enabled systems show potential in educational settings. Lee [20] implemented RAG-based tutors in university STEM fields, enhancing domain alignment, minimizing hallucination, and assisting concept synthesis. In creative fields, Chang et al. [21] showed that RAG could enhance culturally inclusive chatbot design by bolstering factual recall and functional application, though its generative creativity remains limited. Overall, these advancements indicate that RAG not only improves access to information but also serves as a cognitive link between the complexities of biology and design abstraction. When combined with thorough evaluation and systematic diagnostics, RAG–LLM systems present notable possibilities for enhancing both the efficiency and quality of biomimetic design.

Nevertheless, most of these evaluation strategies remain system-centric, emphasizing retrieval precision or error diagnostics. By contrast, the present study foregrounds evaluation at the design outcome level, assessing biological fidelity, semantic clarity, and concept quality through expert judgment. This perspective reflects the interdisciplinary demands of biomimicry design, where retrieval success is ultimately measured by the viability and creativity of resulting concepts, not retrieval accuracy alone. This study also extends recent advances in RAG research beyond NLP into the design domain. For instance, Lewis et al. [2] demonstrated that RAG enhances precision in knowledge-intensive tasks; Majumder et al. [6] showed its ability to generate SAPPhIRE causal models from natural language; and Toukmaji and Tee [7] illustrated how RAG agents could simulate engineer–biologist dialogue for analogical reasoning. Building on these insights, our framework adapts RAG specifically for stage-dependent biomimicry knowledge translation, bridging the Discover–Abstract gap in the BDS.

Beyond the design domain, RAG has also been systematically reviewed in applied domains such as healthcare. Amugongo et al. [22] surveyed the integration of RAG with large language models for clinical knowledge translation, Liu et al. [23] provided systematic guidelines for biomedical applications through meta-analysis, and Gargari and Habibi [24] highlighted opportunities for enhancing medical AI with narrative reviews. These applied-domain studies underscore the broader methodological continuity of RAG: across domains where precision and reliability are critical, evaluation must move beyond retrieval efficiency toward outcome-level assessment. Our study situates biomimicry within this broader methodological landscape, emphasizing that the unique challenges of biological knowledge translation in design parallel those encountered in healthcare. Consequently, this study is directed by three research questions:

RQ1: How does a RAG-LLM system affect the accuracy of biological knowledge translation into design principles?

RQ2: In what ways does the RAG-LLM system support the generation of more relevant and higher-quality design proposals compared to LLM-only use?

RQ3: How do users perceive the contribution of RAG-LLM to the biomimicry design process?

## 3. Materials and Methods

This research employs a RAG methodology within a design framework specifically tailored for industrial design, expanding the application of biological strategies to encompass form language, product semantics, ergonomics, user experience, and brand positioning beyond mere mechanical performance. To organize the creative process, we combine the BDS with the DDM, enabling both systematic exploration and iterative refinement. The BDS outlines six biologically informed phases—Define, Biologize, Discover, Abstract, Emulate, and Evaluate—while the DDM maintains a divergence–convergence rhythm throughout ideation and refinement stages. In this study, the Discover–Abstract phase is not just a functional bottleneck but also a crucial step for aesthetic and semantic interpretation, transforming natural forms and mechanisms into the language of industrial design.

**Figure 1:**
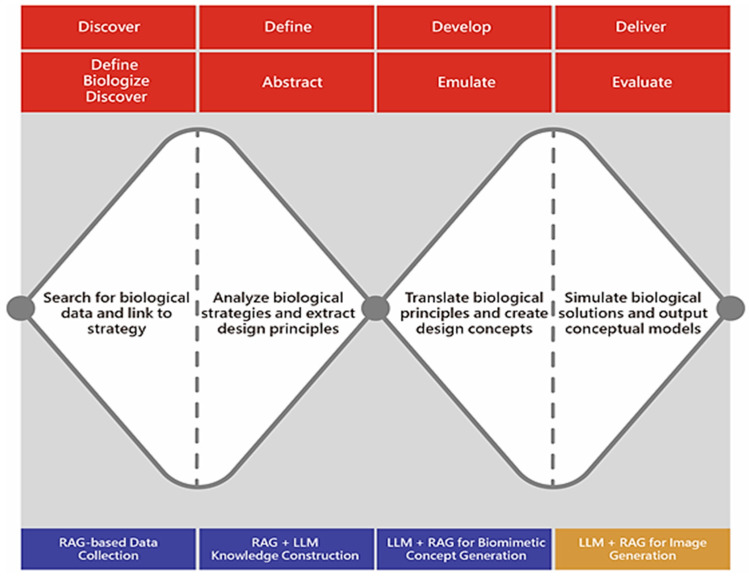
Integrated framework of BDS and DDM. The first diamond (left) aligns with the Define–Biologize–Discover and Abstract stages, while the second diamond (right) corresponds to Emulate and Evaluate. This integration highlights the divergence–convergence rhythm of the DDM combined with the stage-specific guidance of the BDS.

The experimental framework leverages RAG to improve the effectiveness and precision of this translation. By utilizing an LLM linked with curated biomimetic datasets sourced from AskNature, the system retrieves biologically driven references and generates design-oriented textual outputs that detail functional mechanisms along with visual and ergonomic attributes. These outputs directly influence concept sketches, surface treatments, volumetric compositions, and CMF (color–material–finish) decisions in the development of industrial products. Figure 1 showcases this integrated framework. The initial diamond of the DDM (left side) corresponds to the Define–Biologize–Discover–Abstract stages of the BDS, facilitating broad exploration of functional, aesthetic, and semantic opportunities. The subsequent diamond (right side) encompasses the Emulate–Evaluate stages, steering the process toward refined concepts that harmonize biomimetic fidelity, form coherence in industrial design, and user-centric appeal. The RAG system functions within the initial stages of both diamonds, supplying industrial designers with curated biological insights and morphological references, which are then transformed into practical design outputs in the latter stages.

### 3.1. Participants

Two distinct groups were formed for this study. Group One included 30 volunteers aged between 18 and 55 (17 males and 13 females), all from a postgraduate design program and had provided written informed consent. This group’s inclusion required a minimum of one year of design education and knowledge of generative AI. Excluded from this group were individuals with professional biomimicry experience or degrees in natural sciences. Their educational qualifications varied: 6 had bachelor’s degrees, 23 had master’s degrees, and 1 had a doctorate. Group Two consisted of four experienced evaluators (average age of 36.7 years; consisting of 3 men and 1 women) who assessed the generated design images. These evaluators were seasoned design educators and industrial designers with five years of professional practice, and they were not from the student group. They were briefed on the BDS framework and rating criteria before the evaluation. This dual-group approach facilitated a comparative study of how people with different design backgrounds perceive AI-assisted biomimicry. Table 2 presents comprehensive demographic details for both groups.

### 3.2. Apparatus, Software, and Experimental Conditions

The study utilized a high-performance laptop to ensure smooth execution of all software and models without encountering performance issues. The system’s hardware was meticulously selected to meet the LLM and RAG system’s computational requirements. Noteworthy hardware included an Intel^®^ Core™ i9-12900 processor, 48 GB of DDR5 RAM, and an NVIDIA^®^ GeForce RTX™ 3070 Ti Laptop GPU with 8 GB of VRAM for expedited model inference. The main storage was a 1 TB SSD, and detailed specifications can be found in Table 3. The experiment was displayed on the laptop’s 16-inch QHD+ screen with a resolution of 2560 × 1600 pixels and a refresh rate of 165 Hz. Participants used a standard keyboard and mouse to interact with the system. The chief stimulus was a web-based interface facilitating access to one of the three experimental setups: LLM-only, RAG-Small (with a small database), and RAG-Large (with a large database).

**Figure 2:**
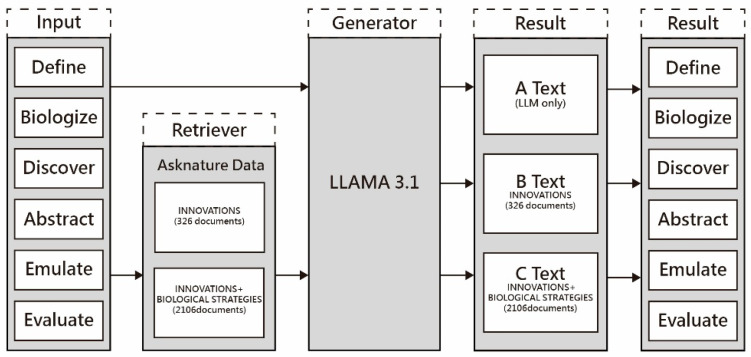
System Architecture of the RAG Application. The framework integrates a curated AskNature knowledge base, a vector database for semantic retrieval, and a locally executed LLM, with outputs connected to evaluation and visualization modules. This architecture illustrates how retrieval and generation components interact to support stage-specific biomimicry design tasks.

In deploying the RAG application framework for this research, the system combined a variety of both local and cloud-based software elements, such as a language model inference engine, embedding models, an interactive user interface, and a containerized environment. Figure 2 illustrates the RAG application infrastructure. This system melds several components from both local and cloud sources, incorporating a language model inference engine, embedded models, interactive interfaces, and a containerized setting. Several essential software elements were utilized in orchestrating this architecture.

Table 4 presents a summary of the main computational tools and their respective versions. The foundation of our language model is Llama 3.1, an open-source model with 8 billion parameters, fine-tuned for instruction and developed by Meta. This particular model was chosen for three key reasons. Firstly, Llama 3.1 excels in conversational tasks and in generating structured responses, making it ideal for language generation focused on design. Secondly, it requires less memory and has lower inference latency than larger models, allowing it to run efficiently on experimental setups with mid-range GPUs (e.g., 8–16 GB VRAM). Lastly, its broad acceptance in the open-source community, as demonstrated by over 96.5 million downloads on the Ollama platform, attests to its reliability and suitability as a baseline model for this study.

The experiment comprised three different conditions:

LLM-only (Non-RAG control)—where responses were solely produced by a locally run LLM (Llama 3.1: 8 b) without the aid of retrieval augmentation.

RAG-Small—this involved the same LLM connected to a vector database created from 326 articles from AskNature Innovations.

RAG-Large—this setup combined the LLM with a database that included 326 Innovations articles as well as 1780 articles on Biological Strategies, totaling 2106 documents.

### 3.3. Experimental Design and Procedure

**Figure 3:**
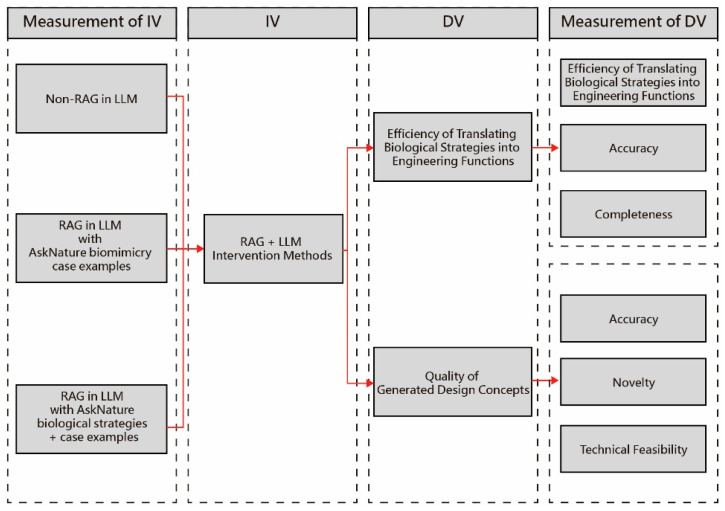
Experimental workflow and relationships between independent variables (IVs) and dependent variables (DVs). The three experimental conditions (LLM-only, RAG-Small, and RAG-Large) feed into a common evaluation pipeline. Each condition generates textual responses across the six BDS stages, with outputs assessed for efficiency, accuracy, completeness, and design concept quality. This workflow highlights how retrieval augmentation influences both knowledge translation and creative outcomes.

The experiment was structured around an automotive design scenario chosen for its familiarity to industrial design students and its relevance in biomimicry case studies (e.g., the Mercedes-Benz Bionic Car). To ensure consistency, prompts were designed for each of the six stages of the BDS (Table 5), and the full experimental workflow is illustrated in Figure 3, including (a) retrieval augmentation, (b) abstraction of biological strategies into design principles, and (c) the generation and evaluation of design concepts. While this study focused on a single design scenario as a proof-of-concept, future research will extend the framework to additional domains and broader participant groups to assess generalizability.

The study employed a quasi-experimental design with three conditions: LLM-only, RAG-Small, and RAG-Large. Generative models were tasked with stimulus creation, while participants acted solely as judges without engaging directly with the models. Performance was evaluated through three dependent variables (DVs): (1) Knowledge Translation Efficiency, measured by mean word count, total generation time, and average time per word during the Define–Abstract stages; (2) Textual Quality, referring to the accuracy and completeness of the descriptions, assessed by two independent evaluators with expertise in biomimetic design using a five-point scale; and (3) Design Concept Quality, covering fidelity to biomimicry, coherence in form, semantic clarity, innovation, user attractiveness, and practical feasibility, rated by four professional designers (average 8.4 years’ experience) on a seven-point Likert scale. The generation of concept images followed a multi-stage Vizcom workflow, detailed in Section 3.5.

The knowledge bases for the RAG conditions were developed utilizing material from the AskNature platform, a publicly accessible and extensively referenced source for biomimicry design [3]. Data collection focused on two main categories: (A) Innovations (326 articles) and (B) Biological Strategies (1780 entries). A custom web scraper, crafted in Python using the requests and BeautifulSoup libraries, was employed to automate data acquisition. This scraper functioned by accessing a predetermined list of target URLs from a text file. For each URL, it methodically traversed the webpage to extract essential data, such as the article title, category, and detailed content. This approach ensured reliable and precise data retrieval. Each complete entry was downloaded as a distinct plain-text (.txt) file, labeled with its original article title for clear identification. The entire dataset was gathered on 18 February 2024. Since the AskNature platform is frequently updated, this dataset captures a specific timeframe. For semantic retrieval relevant to the RAG conditions, this textual knowledge base was converted into a vector index. All 2106 documents were processed with the sentence-transformers/all-MiniLM-L12-v2 model to produce a 768-dimensional vector embedding. These embeddings were stored and indexed using the Facebook AI Similarity Search (FAISS) library. In the experiment, a semantic search employing cosine similarity was conducted against this index to retrieve the top five documents pertinent to a user query. The full text of these five retrieved passages was merged with the user’s initial prompt and fed as enhanced context to the Llama 3.1 LLM. The model operated with a context limit of 2048 tokens and a temperature setting of 0.7 for balancing creativity and determinism in the output generated.

Our RAG framework was utilized in an automotive design case study, seamlessly incorporating specific industrial design factors at every phase of the BDS. Each phase is outlined with corresponding prompts or guiding questions that motivated the AI-driven knowledge interpretation. Table 5 enumerates the prompts employed at each phase of the BDS. The same prompts were provided to all three conditions; only the underlying retrieval approach varied.

Define: The design challenge was framed to address mechanical and functional needs while also taking into account aesthetic identity, ergonomic comfort, and user-centric brand positioning. In the automotive case study, the intention was to visually convey stability, agility, and durability in a unified product language that appeals to the target audience.

Biologize: Functional needs were converted into visual and ergonomic criteria linked to biological analogies, such as distinctive silhouettes, surface patterns, and color indicators relevant to industrial design semantics.

Discover: Pertinent organisms and strategies were identified with a focus on functional mechanics and morphological inspiration. The RAG system selected biological examples known for their unique shapes, textures, and structures that could guide industrial design detailing and CMF selections.

Abstract: Biological strategies were adapted into industrial design principles that blend functional viability with coherent form, symbolic aesthetics, and ergonomic practicality. For instance, rigid exoskeletal references influenced strategies for panel segmentation, surface detailing, and volumetric balance.

Emulate: The abstracted principles were translated into concept designs through sketches and AI-driven image generation, emphasizing surface curves, volume transitions, and areas for user interaction. Functional performance was harmonized with visual aesthetics and styling cues consistent with the brand.

Evaluate: The designs were critiqued for their biomimetic authenticity, semantic precision, user allure, and practical applicability—evaluating not only their functionality but also how effectively they communicated their inspiration to users. This ensured that industrial design values were consistently integrated throughout the process.

### 3.4. Dependent Measures

Performance was appraised using both automated metrics and expert evaluations, offering a comprehensive multidimensional overview consistent with industrial design assessment standards. Automated generation logs and computational analyses evaluated the efficiency and textual characteristics for each experiment:

Information Retrieval Efficiency—Logs recorded the total response time and word count for each prompt. Efficiency was measured as the average time per word generated (total time ÷ word count), allowing for a straightforward comparison across conditions.

Keyword Frequency Analysis—The top 20 most frequent tokens in each condition were identified, with differences in lexical patterns displayed using heat maps.

Inverse Document Frequency (IDF) Analysis—IDF values provided insight into the specificity of biological terms, with higher values indicating rarer, more specialized terminology.

In the qualitative analysis, experts assessed both the quality of Version and design concepts. A rubric evaluated textual quality against four key criteria essential to industrial design: Completeness (ensuring all function elements are present), Accuracy (alignment with validated biological facts), Form Coherence and Semantic Clarity (effective translation of biological forms into a cohesive industrial design language), and Visual Recognizability (clarity of biological inspiration in the concept narrative). However, during initial testing, two professional designers noted that, in practice, textual evaluation is often a holistic process rather than based on distinct criteria. Consequently, a single comprehensive rating per text was used, based on a 7-point Likert scale (1 = very poor, 7 = excellent), with the four criteria serving as guiding considerations. This approach mirrored real-world expert assessments and ensured consistency and ecological validity in the ratings. The text output from the top-performing model was chosen for translation into a visual design concept. To evaluate the quality of the design concept, four industrial design experts assessed the final concept images produced via the Vizcom platform using cue words from the text. Ratings were made on a 7-point Likert scale (1 = strongly disagree, 7 = strongly agree) across three dimensions with two items each, as shown in Table 6.

### 3.5. Image Generation Workflow

Based on the textual quality criteria presented in Section 3.4, the top-performing model’s text was chosen for conversion into a visual design concept. A structured, multi-phase image generation process was crafted and executed, utilizing the Vizcom platform. This process is initiated by creating an initial 2D sketch from the chosen text, using a CAD model of a boxfish to provide a visual framework. Through a series of iterative refinements, including the use of material palettes and the addition of functional elements, the process concluded with a 2D-to-3D transformation to create a photorealistic concept ready for assessment. Details of each workflow stage are found in Section 3.5. The research team opted for the Vizcom platform due to its efficiency in translating complex technical descriptions—such as those produced by the RAG-Large condition—into coherent, high-quality design concepts. Its AI-assisted sketching, palette-based material rendering, and integrated 2D-to-3D transition mimic evolving professional workflows in industrial design, thus underscoring its utility in assessing AI-generated design content. Image generation utilized only outputs from the RAG-Large condition, as these had secured the highest textual quality ratings during preliminary assessments. Essential morphological descriptors, material characteristics, and functional components were extracted from the C-condition texts and incorporated into organized prompts. This ensured that the generated images accurately depicted the biomimetic strategies, stylistic forms, and aesthetic indicators described in the text.

The transition from AI-generated text to assessable design concepts followed a systematic, three-step workflow:

Initial 2D Sketch Generation—This stage began with importing a biologically inspired reference geometry—namely, a side-view model of a boxfish CAD [24]—to set a consistent morphological baseline. Using Vizcom’s Car Shading palette (Vizcom Inc., San Francisco, CA, USA), the model was transformed into multiple 2D concept sketches, focusing on silhouette, proportion, and surface flow while maintaining key biomimetic characteristics.

Refinement with Material Palettes and CAD Primitives—The foundational sketch was refined by applying pre-established material palettes (e.g., matte carbon-fiber exoskeleton, shell-like smooth surfaces) to replicate surface finishes and structural articulation. Functional elements such as windows, wheels, and protective panels were added. Basic 3D primitives derived from a side-view CAD model of the boxfish from the Micro Autonomous Robotic Ostraciiform (MARCO) project (University of Virginia, Charlottesville, VA, USA) were incorporated to enhance volume proportions and structural coherence.

2D-to-3D Conversion and Photorealistic Rendering—The improved 2D sketch was converted into a 3D model using Vizcom’s automatic conversion tool (Vizcom Inc., San Francisco, CA, USA). This stage generated multi-angle inspection views (front, side, isometric) and employed photorealistic rendering to simulate lighting effects, shadows, and environmental contexts (e.g., off-road scenarios). These high-fidelity renderings were integral to the expert evaluation of design concept quality (Section 3.4).

## 4. Results

### 4.1. Analysis of Efficiency and Textual Characteristics

Information Retrieval Efficiency—The efficiency of information retrieval was assessed in different experimental setups: LLM-only (Version A), RAG-Small (Version B), and RAG-Large (Version C). Although the total generation times varied, a closer look at the average time per word showed negligible differences: LLM-only took 0.132 s/word, RAG-Small stood at 0.121 s/word, and RAG-Large at 0.128 s/word, with the greatest variation being only 0.011 s per word (Table 7). With consistent hardware and model settings, these results imply no meaningful divergence in generation efficiency among these methods. This suggests that the data retrieval and integration in the RAG processes do not significantly slow down practical application. While the LLM-only model can start generating outputs immediately, any early speed advantage is neutralized by the energy needed for generating detailed text without retrieval support. Thus, selecting a model for biomimetic design workflows should not prioritize generation speed. Instead, important considerations should be content quality, structured coherence, and semantic accuracy when choosing the most suitable system for these design activities.

**Figure 4:**
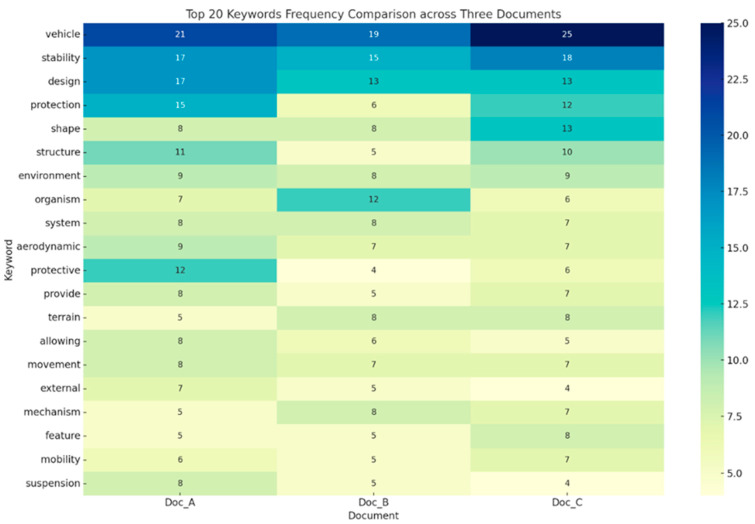
Keyword frequency heatmap of top 20 most frequently occurring terms across all conditions. Darker blue indicates higher relative frequency, while lighter yellow represents lower frequency. RAG-Large produced more domain-specific terms (e.g., “vehicle,” “stability,” “design”), whereas LLM-only relied on generic descriptors. This suggests that RAG enhances terminological precision.

Keyword Frequency Analysis: To evaluate keyword usage among the three texts produced by the different models, the top 20 most frequent terms were identified and displayed in a heatmap (Figure 4). In all three scenarios, terms like “vehicle,” “design,” and “stability” appeared frequently, reflecting a consistent thematic emphasis. The word “protective” was most prominent in the LLM-only text (Version A: 12 instances) but appeared less in RAG-Small (Version B: 4) and RAG-Large (Version C: 6). This indicates that without retrieval enhancement, the model might favor general descriptors. Conversely, Version C featured notably higher occurrences of “shape” (13) and “structure” (10) compared to the others, suggesting that the combination of RAG with an expanded knowledge base prompted the use of vocabulary more closely tied to physical form and morphology.

IDF Analysis of Referenced Organisms: To explore the specificity and commonality of biomimetic inspirations, an inverse document frequency (IDF) analysis was performed on biological terms from the three texts. IDF values measure the rarity of an organism within the corpus, serving as an indicator of each inspiration source’s uniqueness or commonality. Before analysis, we grouped Portuguese man o’ war, Chironex (box jellyfish), and jellyfish under the ‘jellyfish_family’ category to avoid over-fragmentation of related species and ensure accurate trend analysis. The organisms identified were as follows:

Version A (LLM-only): Portuguese man o’ war, jellyfish, armadillo, turtle, shark.

Version B (LLM-RAG-Small, 326 documents): shark, Chironex, pangolin, armadillo.

Version C (LLM-RAG-Large, 2106 documents): boxfish, beetle, turtle, shark.

Seven distinct organism categories surfaced (Table 8). Species appearing in only one text—pangolin, boxfish, and beetle—scored an IDF of 1.0986, showing high specificity. Organisms appearing in two texts—jellyfish_family, armadillo, and turtle—had an IDF of 0.4055, indicating moderate prevalence. The shark, present in all three texts, had an IDF of 0, making it the most common inspiration in the dataset. These results suggest that pangolin, boxfish, and beetle were highly specific biomimetic inspirations, reflecting unique strategies in each text. Jellyfish_family, armadillo, and turtle, while more common, still offered some novelty and distinctive features.

Conversely, the shark was a well-known and frequently applied source, particularly in hydrodynamic drag reduction and structural stability. The organism–text pairings reveal similarities between the pangolin and armadillo, both offering hard protective shells for defense against external threats. This suggests that despite referencing different species, Texts A and B shared similar biomimetic strategies focusing on self-protection and structural defense. Version C included two highly specific species—boxfish and beetle—each with distinct biomimetic properties: the boxfish with its streamlined body and stability, and the beetle with its rigid, lightweight exoskeleton. This diversity indicates a broader range of inspirations in Version C. Considering Version C’s vast knowledge base of 2106 documents used for generation, it is likely that the diversity and originality in its biomimetic strategy stem from the extensive source material. Table 8 summarizes the distribution of organisms and their IDF values across the three conditions, listing each organism, the number of texts it appeared in (N), the degrees of freedom (df), and the corresponding IDF score. Higher IDF values denote a single-text appearance, indicating high specificity, while lower values reflect broader occurrences. These results address RQ1 by demonstrating that RAG-LLM systems enhance biological knowledge translation accuracy in design principles through improved retrieval efficacy and domain-specific terminology utilization.

### 4.2. Text Quality Assessment

To assess the reliability of the text quality evaluations given by the 30 raters, intraclass correlation coefficients (ICCs) were computed utilizing a two-way random-effects model to ensure absolute agreement. Table 9 provides an overview of the outcomes for both single-measure ICC(2,1) and average-measure ICC(2,k). Specifically, it details the ICCs for the evaluations conducted by the 30 raters. The single-measure ICC(2,1) was determined to be 0.213 (95% CI [0.118, 0.400], *p* < 0.001), which suggests a low level of agreement among individual raters. Such a low reliability for single raters is expected in subjective evaluations, as personal biases and interpretive differences can significantly vary (see [25,26]). Conversely, the average-measure ICC(2,k) was found to be 0.890 (95% CI [0.801, 0.952], *p* < 0.001), indicating a high level of reliability when ratings are combined. The study’s design deliberately included a large pool of raters (N = 30) to allow for aggregation, effectively reducing individual bias and random error, thus yielding stable consensus scores [27]. Consequently, the analysis relies on averaged ratings rather than individual ones, ensuring reliable measurements despite variations among raters.

Table 10 illustrates the descriptive statistics for textual quality assessments throughout the six design phases: Define, Biologize, Discover, Abstract, Emulate, and Evaluate, for the three experimental versions A, B, and C. Version C regularly obtained the highest average scores across most phases, notably in Discover (M = 5.957, SD = 1.008) and Abstract (M = 5.778, SD = 1.257). Version A usually placed second, demonstrating notable results in Define (M = 5.266, SD = 1.099) and Biologize (M = 5.422, SD = 1.100), whereas Version B often received the lowest scores, particularly in Define (M = 4.244, SD = 0.747) and Abstract (M = 4.243, SD = 0.986). The 95% confidence intervals display little overlap between the highest and lowest-rated versions in several stages, indicating notable differences in perceived textual quality among the versions.

**Figure 5:**
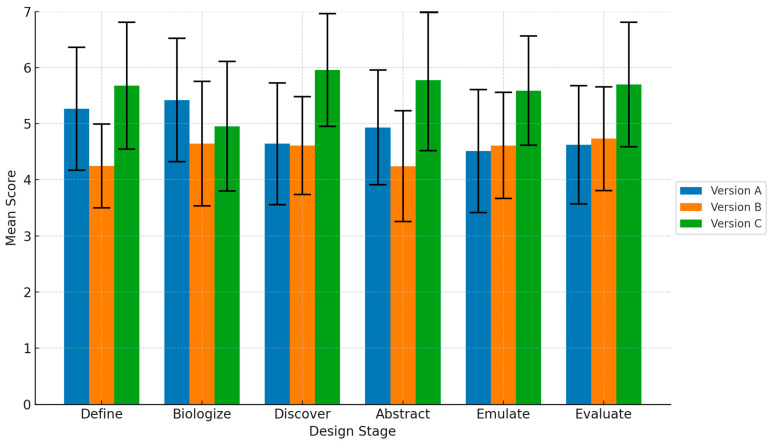
Comparison of mean scores across six BDS stages (Define–Evaluate). Error bars show standard deviations. RAG-Large outperformed other conditions, particularly during cognitively demanding stages such as Abstract and Emulate. This indicates that retrieval support is most beneficial in bridging the Discover–Abstract bottleneck.

In Figure 5, the mean scores of the three versions throughout the six design stages are depicted, with error bars indicating standard deviations. This figure reflects the overall trend noted in the descriptive statistics (Table 1), revealing that Version C consistently received higher average ratings compared to Versions A and B, especially in the later stages (Discover, Abstract, Emulate, and Evaluate), whereas Version B frequently recorded the lowest ratings.

To systematically evaluate these differences, one-way ANOVAs were performed at each stage to compare the three versions (A, B, and C). The results indicated significant overall differences across all stages: Stage 1 (F(2, 87) = 16.082, *p* < 0.001), Stage 2 (F(2, 87) = 3.647, *p* = 0.030), Stage 3 (F(2, 87) = 17.901, *p* < 0.001), Stage 4 (F(2, 87) = 14.749, *p* < 0.001), Stage 5 (F(2, 87) = 10.478, *p* < 0.001), and Stage 6 (F(2, 87) = 9.886, *p* < 0.001) (Table 11). Subsequent Tukey HSD post hoc tests revealed that Version C significantly outperformed Versions A and B in most of the later stages, while differences between Versions A and B were more varied. These outcomes confirm the descriptive trends, emphasizing Version C’s superiority in enhancing creative synthesis and evaluation in the final stages of the design process.

Table 12 presents the results of the Tukey HSD test for pairwise comparisons among the versions at each stage. Notably, significant differences appeared most often between Version C and the other versions, especially in the later stages. In contrast, variations between Versions A and B were less consistent.

In summary, the findings indicate that Version C frequently outperforms Versions A and B, particularly during the latter stages of the design process, while the differences between Versions A and B are less predictable. These performance trends imply that the design strategy of Version C offers benefits in enhancing creative synthesis and evaluation in the later stages, a topic further elaborated in the Discussion section. This result addresses RQ2, showing that RAG-LLM enhancement aids in producing more pertinent and superior design proposals compared to using only LLM.

### 4.3. Design Image Generation and Quality Assessment

**Figure 6:**
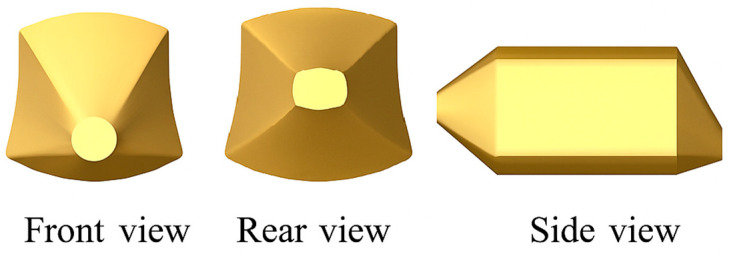
CAD models of Spotted boxfish, used as biological references. The streamlined morphology provided a baseline for evaluating AI-generated automotive concepts. This exemplar highlights how biological form can inform structural coherence and aerodynamic design. (redrawn from [28], p. 111).

Initially, this research synthesized the conclusions derived from the C-Version throughout each phase of the biomimetic design process. By implementing RAG, we asked: “If we were to create an image based on this conclusion, what prompt would we use?” The model-generated responses served as foundations for image generation prompts, ensuring they accurately captured the essential concepts and morphological aspects of biomimetic design. The final prompt integrated biological configuration elements from the text with automotive styling terminology, allowing modifications for various design stages: “Create a futuristic, biomimetic off-road adventure vehicle inspired by the cubic shape of the boxfish and the shell of a sea turtle. The vehicle features a robust carbon fiber exoskeleton with a smooth, matte finish, merging natural elements with advanced engineering. Its streamlined sections enhance aerodynamics, facilitating efficient, high-speed travel. The design highlights adaptability to advanced engineering and showcases its inspiration drawn from the environment.” Following the design direction outlined in Version C, and using the boxfish as the main reference for the vehicle outline, we chose a CAD side-view model of the boxfish from the Micro Autonomous Robotic Ostraciiform (MARCO) [28]: Hydrodynamics, Design, and Fabrication for the initial modeling reference in image generation. This CAD image offered a morphologically consistent standard for vehicle styling concepts (Figure 6).

**Figure 7:**
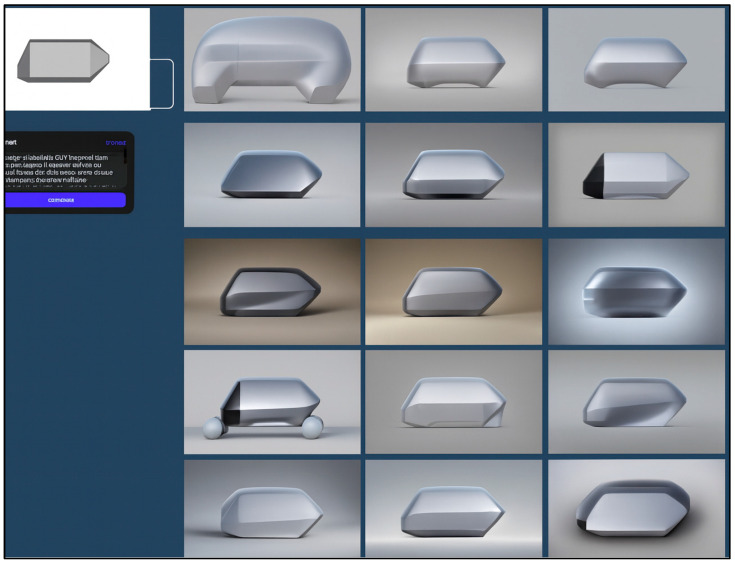
Phase 1 of concept generation: Basic image outputs derived from a boxfish CAD side-view silhouette using Vizcom’s “Car Shading” palette. Initial sketches emphasized overall proportion and surface flow, guided by aerodynamic and stability-focused prompts. This stage illustrates how biological morphology was visually translated into early-stage design proposals.

**Figure 8:**
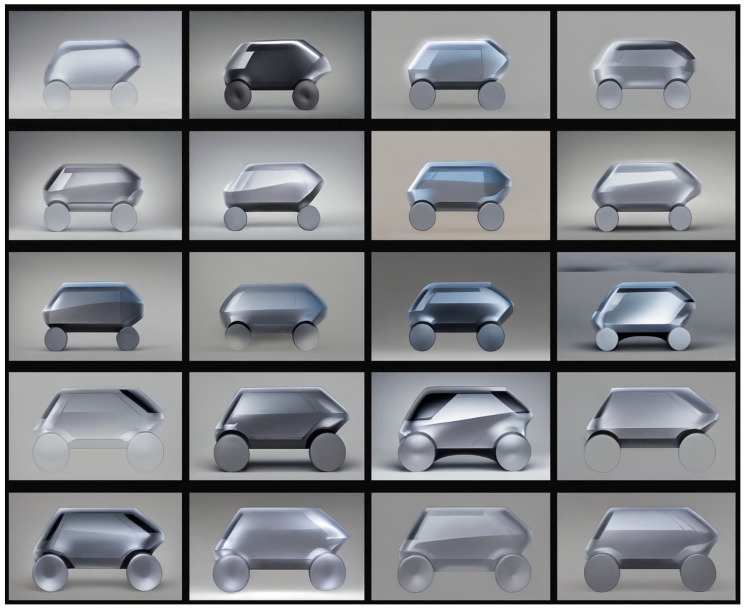
Phase 2 of concept generation: Integration of functional components (e.g., wheels, windows, structural panels) into preliminary sketches. These additions demonstrate how functional analogies from biology were embedded into evolving design concepts.

**Figure 9:**
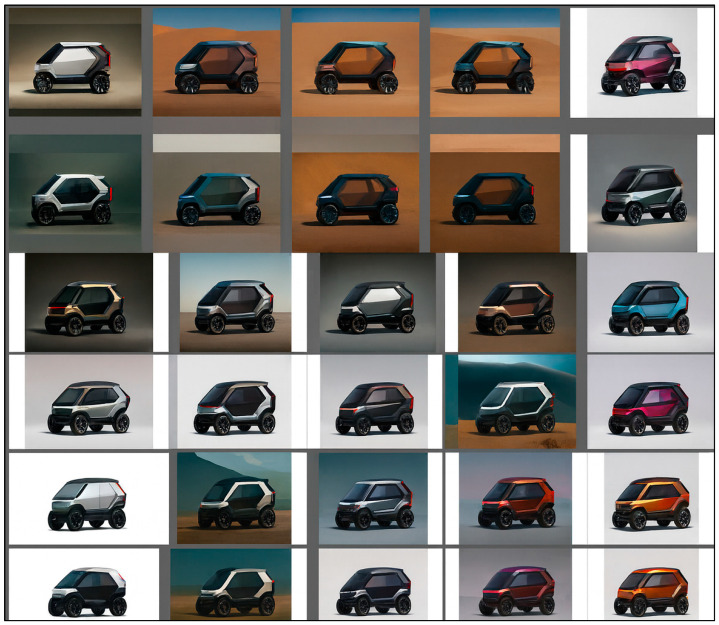
Phase 3 of concept generation: Enhanced design details emphasizing structural and aesthetic alignment with biological inspirations. Notably, surface segmentation was guided by beetle exoskeleton analogies, reinforcing functional and stylistic fidelity.

Three-Step Image Generation Process: Building on the previously outlined workflow from AI-generated text to assessable design concepts, the image generation was divided into three successive phases (Figure 7, Figure 8 and Figure 9): (1) Initial image generation—exploration of biomimetic form language; (2) Functional component integration—ensuring practical feasibility; (3) Design detail enhancement—achieving presentation-level visual quality.

Phase 1: Initial Image Generation—A side-view silhouette of a boxfish in CAD was transformed into multiple 2D body design sketches using Vizcom’s Car Shading palette. Prompt weighting was modified to translate the boxfish’s natural morphology into variations reflecting industrial design language, focusing on streamlined flow, grounded stability, and volumetric balance. The guiding prompt specified smooth, continuous surfaces, slightly tapered edges, horizontal proportions, and gentle curves to indicate aerodynamic efficiency and a low center of gravity (Figure 7).

Phase 2: Integration of Functional Components—Key structural elements, such as windows and wheels, were manually incorporated into the outputs from Phase 1 to guarantee functional practicality. Enhancements to proportions, structure, and style were achieved using the Car Shading palette in Vizcom, as illustrated in Figure 8. At this point, dependency on LLM/RAG content was low, indicating a transition from conceptual exploration to focusing on functional feasibility.

Phase 3: Detailed Design Enhancement—The designs from Phase 2 underwent further refinement by incorporating automotive details such as headlights, air intakes, and rearview mirrors, as well as surface finishes, utilizing Vizcom’s Realistic Product and Exterior palettes. Descriptive features from Version C, like “sturdy carbon fiber exoskeleton,” “smooth surface,” and “matte finish,” were included to synchronize visuals with the biomimetic narrative. Numerous iterations with diverse style influence settings resulted in high-fidelity concept sketches suitable for presentations (Figure 9). Table 13 provides an overview of the inputs, tools, AI utilization levels, and outputs across each phase. It emphasizes the transition from significant AI reliance during conceptual exploration (Phase 1) to minimal AI involvement in functional integration (Phase 2), followed by moderate AI assistance in visual refinement (Phase 3).

**Figure 10:**
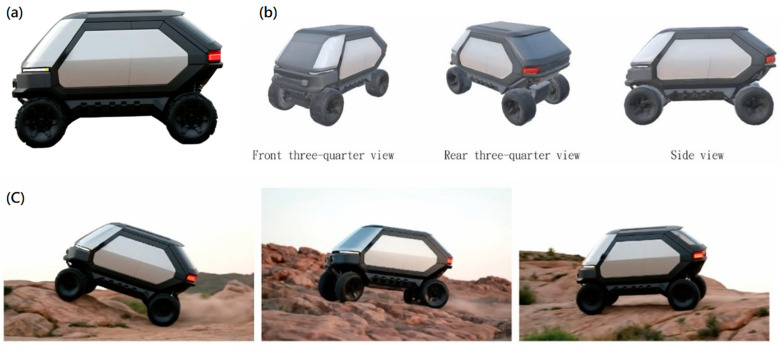
2D-to-3D model conversion and contextual visualization of the biomimetic vehicle. Vizcom-generated 3D models are shown from multiple perspectives: (**a**) front view and (**b**) side/isometric views. Panel (**c**) places the model in AI-generated backgrounds to support structural verification, scenario simulation, and design presentation. This stage demonstrates the feasibility of translating biological principles into manufacturable product semantics.

3D Modeling and Contextual Visualization: To enhance the evaluation of three-dimensional visual quality and application, the ultimate 2D sketches were transformed into 3D models utilizing Vizcom’s integrated model generation feature (Figure 10). This facilitated the validation of structure and proportions from various viewpoints (front, side, and isometric) and enabled adjustments to align with design objectives. Additionally, Vizcom’s environmental integration capabilities were employed to place models into real-world scenarios and produce brief video clips (e.g., on rocky terrain at dusk). These engaging outputs aided in design presentations, concept validation, and expedited decision-making.

**Figure 11:**
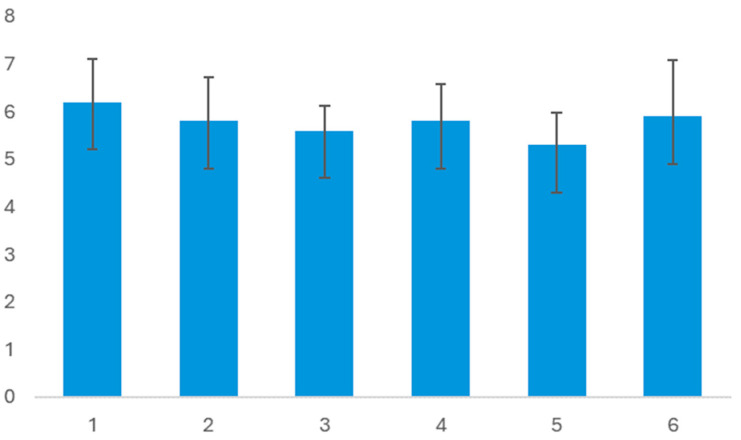
Comparison of mean scores of the six evaluation criteria for design quality (fidelity, discernibility, novelty, stylistic alignment, innovation, and potential to challenge conventions). Error bars represent standard deviations. RAG-Large achieved the highest ratings across most criteria. For example, beetle-inspired exoskeletal structures were rated highly for innovation and functional coherence.

Evaluation of Design Quality: The design quality scale, comprising six items, exhibited moderate internal consistency (Cronbach’s α = 0.67), which is suitable for exploratory studies with small samples and multi-dimensional constructs evaluated with few items per dimension [29,30]. The reliability of the design quality ratings was assessed using a two-way random effects intraclass correlation coefficient [ICC(2,k)] with four raters. Results indicated a moderate degree of consistency for single raters (ICC = 0.440, 95% CI = 0.038–0.865), suggesting that individual ratings alone may not provide sufficiently stable estimates. However, when the ratings were averaged across the four raters, reliability improved to a good level (ICC = 0.759, 95% CI = 0.137, 0.962) [26], indicating that aggregated ratings yield a more dependable assessment of design quality. The associated F-test confirmed that the observed agreement among raters was significantly greater than chance, F(5,15) = 4.143, *p* = 0.015 (Table 14). Table 15 depicts the descriptive statistics for questionnaire items. These findings suggest that, while individual rater judgments are variable, the consensus ratings provide a robust basis for evaluating design quality in biomimetic design tasks. Figure 11 illustrates the mean score comparison for the six criteria. These findings pertain to RQ3, offering insights into users’ views on the impact of RAG-LLM in the biomimicry design process. Overall, the results address RQ1–RQ3 by showing enhancements in knowledge translation accuracy, design proposal quality, and user perception of the RAG-LLM method.

## 5. Discussion

This discussion, guided by our trio of research inquiries, assesses how the RAG-LLM system (RQ1) advanced the translation of biological knowledge, (RQ2) elevated the quality of design proposals, and (RQ3) influenced user perspectives on its role in the biomimicry design process.

### 5.1. Summary of Key Findings

This research assessed the effectiveness of an AskNature-based RAG framework integrated with an LLM, catering to two pivotal phases in the biomimicry design process: (1) converting biological strategies into explicit, biologically informed text, and (2) crafting corresponding visual design concepts. Evaluations of both text and imagery demonstrated strong adherence to biomimicry principles, alignment with intended design directions, and successful communication of functional analogies. These outcomes support previous calls for computational approaches to enhance knowledge transfer in biomimetic design [4,26], along with recent findings that AI-aided retrieval enhances the cross-domain interpretation of specialized knowledge [31].

Cumulatively, these results directly address RQ1, showing that the RAG-LLM system enhances the accuracy and clarity of translating biological knowledge, and RQ2, indicating its role in producing superior design proposals that convey analogical reasoning with greater efficacy. They also relate to RQ3, as differences in evaluators’ assessments illustrated that users perceived the system’s outputs as both credible and open to varied interpretations. The tri-phase image-generation process exhibited how AI could achieve a balance between conceptual fidelity and stylistic diversity, while the textual outputs offered specificity and rich semantic detail.

Theoretically, this supports the assertion that multi-modal AI has the potential to close the enduring “translation gap” between biological insights and design ideation [32]. Measurements noted differences in evaluator judgments that highlight a recurring challenge in creative evaluation: interpretations are swayed by disciplinary backgrounds, cognitive preferences, and the varied weighting of evaluation criteria [31,33]. While this variability introduces measurement challenges, it also exemplifies the inherent complexity in assessing design quality.

### 5.2. Comparison with Prior Research and Theoretical Implications

The assessment of reliability for both text and design evaluations showed notably low single-measure intraclass correlation coefficients (ICCs), whereas the average-measure ICCs suggested moderate reliability, indicating considerable divergence among individual raters that nonetheless resulted in a consistent group consensus. For design quality, the ICC(2,1) = 0.31 denoted low reliability for individual raters, in contrast to ICC(2,k) = 0.64, which suggested a moderate reliability for an average of four raters [22,23,24]. Similar patterns were evident in text quality ratings, where individual agreements were weak but greatly improved when averaged. Structured rubrics are acknowledged for reducing, though not entirely eliminating, variability among raters in evaluating creative work [34,35]. This ongoing variance is due to the complexity of design quality, which defies simplification to a single criterion [36], and raters’ use of distinct cognitive frameworks [32].

Our findings extend these insights to AI-assisted design, illustrating that while algorithms can guide evaluations, they cannot replace subjective judgment. The internal consistency of the design quality scale (Cronbach’s α = 0.67) and similar observations for text quality align with psychometric standards, as constructs covering multiple subdimensions with limited items per dimension often lead to underestimated α values [29,37]. This range was intentional, aiming to encompass aspects like conceptual integrity, structural creativity, and clarity in communication, each significant in different settings. Theoretically, these results highlight the significance of consensus or weighted aggregation methods [26,30,38] to enhance reliability without disregarding evaluative diversity. The findings answer RQ3: participants appreciated the RAG-LLM system as an effective mediator in biomimicry design, though their assessments remained influenced by disciplinary frameworks and individual preferences. AI shapes the perception of design quality but does not remove the element of subjectivity.

Prior evaluation advances such as Mindful-RAG [18] and eRAG [19] highlight system-level performance by reducing hallucinations and improving retrieval metrics. Our study extends this perspective by situating evaluation within the design process itself, where effectiveness must be judged not only by factual accuracy but also by the creativity, functional translation, and biological coherence of the outputs. This design-centered evaluation approach underscores that while retrieval reliability is foundational, domain-specific assessments are essential for capturing the interdisciplinary success of biomimicry innovation. These findings parallel observations from healthcare RAG reviews [22,23,24], which emphasize that rigorous evaluation requires moving beyond retrieval accuracy to outcome-level measures that reflect domain-specific utility. Just as medical applications assess clinical relevance and decision-support reliability, our design-focused framework foregrounds biological fidelity, creativity, and concept quality as outcome-oriented criteria for evaluating RAG systems. Overall, the results address RQ3, indicating that participants viewed the RAG-LLM system as a valuable intermediary in biomimicry design. However, their evaluations were still impacted by their disciplinary backgrounds and personal tastes. AI influences how design quality is perceived, but it does not eliminate subjective judgment.

While the IDF analysis provided measurable evidence of retrieval diversity and specificity, it should be interpreted cautiously. Higher IDF values indicate less frequent and therefore more distinctive organisms, but this does not ensure ecological appropriateness or biological correctness. A species may appear statistically rare in the dataset yet still be irrelevant or misleading for the intended design task. Future work should therefore incorporate ecological or biological expert validation, cross-database comparisons, and triangulated evaluation methods to ensure that retrieval diversity aligns with biological plausibility and design relevance.

### 5.3. Alignment with Broader AI-Assisted Design Research

The impressive results achieved by the large-scale RAG configuration align with recent research illustrating that enhancing LLMs with domain-specific retrieval significantly boosts feasibility, innovation, and semantic accuracy in design ideation [7]. Our findings indicate that the advantages of RAG are most evident during later-stage, cognitively intensive phases—like abstraction, emulation, and evaluation—where precise analogical thinking and contextual synthesis are crucial. To the best of our knowledge, this study is the first to explicitly integrate the DDM’s Define and Develop stages with the BSD while incorporating a RAG-enhanced LLM. This integration forms a two-tier dynamic model: RAG facilitates the retrieval and translation of biological knowledge, allowing for quick iterations between macro-level framing and micro-level solution crafting. Such a stage-dependent support framework enhances design theory by detailing the timing and methods for the most effective AI interventions. Our stage-level findings also highlight that not all phases receive equal benefits. The Define and Biologize stages, which primarily focus on information gathering, showed smaller performance differences between RAG and baseline LLMs, implying that the core capabilities of LLMs might suffice for early-stage, less complex tasks. On the other hand, phases requiring complex integration and synthesis saw significant advantages from RAG’s improved retrieval abilities. These stage-specific effects elucidate the system’s response to RQ1 and RQ2: early knowledge discovery tasks benefited less from RAG, but abstraction and emulation phases in later stages demonstrated notable improvements in the quality of proposals and semantic precision. This pattern also relates to RQ3, as evaluators found the system most beneficial when cognitive demands were at their peak.

### 5.4. Practical and Theoretical Implications

The RAG-LLM setup pragmatically offers a scalable method to tackle the longstanding issue in biomimicry—the transition from discovery to abstraction—by providing relevant analogies and systematic translations of biological concepts. This approach eases the cognitive demands on designers who lack formal biology education and improves the traceability of their design decisions. On a theoretical level, our results support the notion that creative evaluations in AI-aided settings should employ consensus-driven methodologies to accommodate the variability inherent in subjective assessments. Approaches like rater calibration, iterative refinement of evaluation criteria, or expertise-weighted scoring [37] may enhance reliability while preserving interpretive depth. Additionally, the suggested dynamic three-stage AI integration framework—covering data acquisition, idea generation, and iterative refinement—illustrates how AI can align the exploratory–convergent cycles of the DDM with the iterative micro-loops of the BSD.

### 5.5. Limitations and Future Directions

This study is limited by its relatively small sample size (30 postgraduate students) and focus on a single automotive design task. These constraints mean that the findings should be interpreted as exploratory and proof-of-concept rather than definitive generalizations.

Another limitation is that the participant pool consisted solely of design students without formal biological training. This background may bias evaluations toward usability and clarity of design outcomes rather than the ecological or biological accuracy of abstractions. Furthermore, the evaluation pipeline relied heavily on Likert-scale ratings, which—though structured and widely applied—remain inherently subjective. Future studies should therefore involve participants with biological expertise to complement design-focused judgments, and adopt more objective evaluation metrics (e.g., semantic similarity measures, ecological validation) to triangulate findings. Broader investigations will also expand the participant base to include diverse cohorts (e.g., design and non-design students, professional designers) and extend evaluation to multiple design domains, including consumer electronics and healthcare, thereby providing stronger evidence for the framework’s generalizability.

Beyond participant and evaluation constraints, technical limitations must also be acknowledged. While the AskNature database is considered authoritative, it comprises a limited number of entries (approximately 2118), restricting the diversity of information retrieval. Expanding to include broader, cross-disciplinary, and multilingual databases could enhance the range of creative inspiration. Technically, constraints on local execution required the use of smaller-scale language models, which might underperform compared to large-scale cloud-based models. Similarly, image generation was conducted using a generic online platform with limited control over style; future investigations could consider specialized multimodal models that are fine-tuned with domain-specific design datasets. Further research should aim to identify the optimal balance between retrieval diversity and relevance, incorporate diverse inspiration sources (such as visual and auditory), and assess the AI-assisted methodology across various design fields. Comparative studies could analyze the impact of different language model architectures, RAG configurations, and evaluation frameworks to develop more refined AI support strategies specific to each design phase.

## 6. Conclusions

This study directly tackles the addresses the challenge to enhance the conversion of biological strategies into practical and innovative design ideas during the important Discover–Abstract stage in the BDS. We illustrated that a RAG framework—merging LLMs with a curated, specialized biomimetic knowledge base—can provide design-ready insights that cater to the tangible requirements of designers, even those lacking expertise in biology. By synchronizing the BDS with the DDM, the suggested framework offers a transparent and repeatable route from biological inspiration to conceptual design. The inclusion of T2I visualization and 2D-to-3D conversion promotes quick, iterative advancement, improving both adherence to biological principles and the richness of design outcomes. This organized method shifts AI from being a simple information repository to an approachable design partner, boosting design fluency and basing creative choices on scientifically validated principles. Regarding our research questions, the results indicate that (RQ1) the RAG–LLM augmentation increased retrieval efficiency and improved textual accuracy, (RQ2) it elevated the quality and precision of biomimetic design ideas, and (RQ3) it supported evaluative processes without removing subjectivity, as users’ disciplinary frameworks continued to influence their interpretations. These findings collectively highlight both the possibilities and constraints associated with AI-assisted biomimicry design. In addition to practical applications, the research enhances theoretical understanding of AI-driven biomimetic design by showing how strategic RAG integration can accelerate innovation while maintaining biological precision. Future endeavors should investigate extending the framework to encompass multimodal and cross-domain knowledge bases, adaptive user interfaces, and dynamic retrieval–generation techniques. These advancements could fortify the link between biology and design, spreading the advantages of AI-supported biomimicry across a broader spectrum of industries and educational fields.

## Figures and Tables

**Table 1:** Comparison of three representative biomimicry design models (BDS, BioTRIZ, and SAPPhIRE/BIDARA). While BioTRIZ and SAPPhIRE provide systematic reasoning, they require specialized expertise, whereas the BDS is more intuitive but limited by manual retrieval in the Discover–Abstract stage. This highlights the rationale for integrating BDS with AI-supported tools.

Model	Knowledge and Process	Engineering Focus	Strengths	Limitations/Gaps	References
BDS	Stepwise process (Define →Biologize→Discover →Abstract→Emulate→Evaluate) translating biological strategies into design concepts	Broad applicability across product and system design; emphasizes sustainability and innovation	Intuitive and iterative; suitable for general design practice	Low computational support; inefficient knowledge retrieval; Discover–Abstract transition depends on biological expertise	[3,8,13]
BioTRIZ	TRIZ-based contradiction-solving mapped to biological analogies	Functional optimization and engineering conflict resolution	Structured engineering reasoning; effective for systems	Requires TRIZ expertise; limited support for complex biological systems	[9,10]
SAPPhIRE/BIDARA	Causal model (System–Action–Part–Phenomenon–Input–oRgan–Effect) for representing biological mechanisms	Computational reasoning; supports knowledge-based engineering and automated concept generation	Formalized knowledge modeling; scalable for database integration	High modeling complexity; requires detailed biological data; less intuitive for non-engineers	[6,11]

**Table 2:** Demographic characteristics of participants in the version comparison and image quality evaluation experiments. The sample included 30 postgraduate industrial design students, with a balanced mix of genders and varying levels of design background and AI experience. These characteristics provide context for interpreting evaluation outcomes and highlight the exploratory nature of the study.

Category	Subcategory	Version Comparison Experiment (n)	Percentage (%)	Image Quality Evaluation (n)	Percentage (%)
Gender	Male	17	56.7	3	75.0
	Female	13	43.3	1	25.0
Education	Bachelor’s degree	6	20.0	0	0.0
	Master’s degree	23	76.7	3	75.0
	Doctoral degree	1	3.3	1	25.0
Age	18–25 years	19	63.3	1	25.0
	26–35 years	10	33.3	2	50.0
	36–55 years	1	3.3	0	0.0
	Above 55 years	0	0	1	25.0

**Table 3:** Hardware specifications of the experimental system used for model execution and evaluation. The setup included a consumer-grade laptop with an RTX 4050 GPU, ensuring consistent performance across all conditions. These specifications contextualize the feasibility of running retrieval-augmented generation workflows on accessible, mid-range hardware rather than high-performance clusters.

Component	Specification
Processor (CPU)	Intel^®^ Core™ i9-12900 (14 cores/20 threads)
Graphics Card (GPU)	NVIDIA^®^ GeForce RTX™ 3070 Ti Laptop GPU/8 GB, GDDR6 VRAM, 120 W TGP
Memory (RAM)	48 GB DDR5-4800

**Table 4:** Computational tools of the experimental system. The setup combined Python-based environments (e.g., Jupyter Notebook 7.0.8, Colab) with AI-specific platforms (Ollama, AnythingLLM, Vizcom) to enable retrieval, generation, and visualization. These tools ensured an integrated workflow for implementing and evaluating the RAG-based biomimicry design framework.

Computational Tools	Version
Python	3.11.5
LLM	Llama 3.1:8b
Ollma	0.5.11
OpenWeb UI	V0.6.4
Docker	4.38.0
Llama	3.1

**Table 5:** Seven-point rubric used to evaluate RAG-generated vehicle design images. The rubric covered six key criteria: fidelity to biomimicry, discernibility of biological inspiration, novelty of design, stylistic alignment with intended imagery, innovation of structures or functions, and potential to challenge conventional vehicle concepts. This structured evaluation framework enabled expert raters to assess both functional adherence and creative originality in the generated designs.

Stage	Prompt Brief	Example Wording
Define	Frame the design problem	How can a vehicle be designed to adapt to extreme off-road exploration environments while ensuring stability, mobility and protection? How can rigid structures and aerodynamic characteristics enhance maneuverability?
Biologize	Identify relevant organisms and structures	Which organisms have square-like shapes with good stability and aerodynamic properties? Which organisms possess adaptive external protective mechanisms?
Discover	Retrieve biological strategies and precedents	Which organisms have been proven to influence fluid dynamics through their rigid frameworks? Which of these have already inspired vehicle design concepts in previous research?
Abstract	Translate biology into engineering principles	How can the identified strategies be translated into vehicle body design to ensure stability and adaptability? How can exoskeletal structures influence aerodynamics and be integrated with space-utilization design?
Emulate	Integrate principles into a concept	How can rigid exoskeletal structures, aerodynamic shapes and adaptive protective mechanisms be integrated to develop an initial off-road exploration vehicle design? What prototyping techniques (CAD simulations, materials, structural testing) can validate these concepts?
Evaluate	Assess feasibility and refine	Can the biomimetic design achieve stability, mobility and protection in real environments? How do materials, structures and fluid dynamics compare to natural systems? What manufacturing or commercial challenges might arise, and which stage of the BDS should be revisited to improve maturity?

**Table 6:** Seven-point rubric for evaluating RAG-generated vehicle design images. The rubric included six criteria: (1) adherence to biomimicry, (2) discernibility of the biological inspiration, (3) novelty of the design, (4) stylistic alignment with intended imagery, (5) innovation of structures or functions, and (6) potential to challenge conventional vehicle concepts. This structured scale enabled expert raters to assess both functional adherence and creative originality in the generated concepts.

Criterion (Label)	Rater-Facing Item
Adherence to the bionic concept	1. Whether the design adheres to the bionic concept?
Discernibility of the inspiration	2. Whether the bionic inspiration is clearly discernible in the design?
Novelty of the design	3. Whether the design is novel?
Stylistic alignment with intended imagery	4. Whether the design style matches the image?
Innovation in structure or functionality	5. Whether the vehicle exhibits innovative structure or functionality?
Potential to challenge conventional vehicle concepts	6. Whether it offers a novel concept of vehicle design?

Note. All items are rated on a 7-point Likert scale (1 = strongly disagree, 7 = strongly agree).

**Table 7:** Generation speed across experimental conditions (LLM-only, RAG-Small, and RAG-Large). Metrics include mean word count, total generation time, and average time per word during the Define–Abstract stages. Results show that retrieval augmentation (RAG-Small and RAG-Large) did not significantly reduce generation efficiency compared to LLM-only, while producing more information-rich outputs.

Mode	Content Length	Total Generation Time	Average Time per Word
A (LLM-only)	2227 words	294 s	~0.132 s/word
B (RAG-Small)	1900 words	231 s	~0.121 s/word
C (RAG-Large)	2029 words	260 s	~0.128 s/word

**Table 8:** IDF values of organisms appearing in the three Version ConditionsInverse document frequency (IDF) values of organisms retrieved across the three experimental conditions (LLM-only, RAG-Small, and RAG-Large). Higher IDF values indicate rarer and more specific inspirations (e.g., pangolin, boxfish, beetle), while lower values denote commonly retrieved organisms (e.g., shark). The results show that RAG-Large produced more diverse and unique inspirations, reflecting its broader knowledge base, whereas LLM-only leaned on familiar species. These findings highlight how RAG improves both diversity and specificity in biomimetic knowledge translation.

Item No.	N	df	IDF
1	boxfish	1	1.0986
2	pangolin	1	1.0986
3	beetle	1	1.0986
4	jellyfish_family	2	0.4055
5	armadillo	2	0.4055
6	turtle	2	0.4055
7	shark	3	0

**Table 9:** Intraclass correlation coefficients (ICCs) for text quality ratings (N = 30). A two-way random-effects model with absolute agreement was used. Single-measure ICC(2,1) values indicated low agreement among individual raters (ICC = 0.213, 95% CI [0.118, 0.400]), while average-measure ICC(2,k) values demonstrated high reliability when ratings were aggregated (ICC = 0.890, 95% CI [0.801, 0.952]). These results suggest that although individual judgments varied considerably, consensus ratings across multiple raters provided a stable and reliable assessment of text quality.

Measure type	ICC	95% CI (Lower, Upper)	*F*	df1	df2	*p*
Single measures (ICC[2,1])	0.213	0.118, 0.400	11.063	16	464	<0.001
Average measures (ICC[2,k])	0.890	0.801, 0.952	11.063	16	464	<0.001

Note. ICC values are based on a two-way random-effects model, absolute agreement, average measures (ICC[2,k]). Interpretation follows [23] guidelines: values ≥ 0.75 indicate good agreement, and values ≥ 0.90 indicate excellent agreement.

**Table 10:** Descriptive Statistics for Each Design Stage by Version (N = 30).

Design Stage	Text	Mean	SD	95% CI (Lower, Upper)
Define	A	5.266	1.099	4.856, 5.677
	B	4.244	0.747	3.965, 4.523
	C	5.678	1.132	5.255, 6.101
Biologize	A	5.422	1.100	5.011, 5.833
	B	4.644	1.111	4.229, 5.059
	C	4.954	1.157	4.522, 5.386
Discover	A	4.644	1.086	4.238, 5.050
	B	4.610	0.870	4.285, 4.935
	C	5.957	1.008	5.580, 6.333
Abstract	A	4.932	1.022	4.550, 5.314
	B	4.243	0.986	3.875, 4.612
	C	5.778	1.257	5.308, 6.247
Emulate	A	4.511	1.096	4.101, 4.920
	B	4.611	0.947	4.257, 4.965
	C	5.589	0.974	5.225, 5.953
Evaluate	A	4.622	1.055	4.228, 5.016
	B	4.733	0.924	4.388, 5.078
	C	5.700	1.109	5.286, 6.114

**Table 11:** Analysis of variance (ANOVA) results for text quality across the six stages of the BDS. The analysis compares performance across the three conditions (LLM-only, RAG-Small, and RAG-Large). Significant differences emerged in the Abstract and Emulate stages, where RAG-Large outperformed the other conditions. These results indicate that retrieval augmentation is particularly effective in supporting stages that require higher levels of knowledge translation and creative synthesis.

		Sum of Squares	df	Mean Square	F	Sig.
Stage 1	Between Groups	32.684	2	16.342	16.082	<0.001
	Within Groups	88.404	87	1.016		
	Total	121.088	89			
Stage 2	Between Groups	9.195	2	4.598	3.647	0.030
	Within Groups	109.689	87	1.261		
	Total	118.884	89			
Stage 3	Between Groups	35.277	2	17.638	17.901	<0.001
	Within Groups	85.722	87	0.985		
	Total	120.999	89			
Stage 4	Between Groups	35.388	2	17.694	14.749	<0.001
	Within Groups	104.370	87	1.200		
	Total	139.758	89			
Stage 5	Between Groups	21.277	2	10.638	10.478	<0.001
	Within Groups	88.333	87	1.015		
	Total	109.610	89			
Stage 6	Between Groups	21.084	2	10.542	9.886	<0.001
	Within Groups	92.774	87	1.066		
	Total	113.858	89			

**Table 12:** Tukey HSD post hoc comparisons between experimental conditions (LLM-only, RAG-Small, and RAG-Large) for each stage of the BDS. Significant pairwise differences were primarily observed in the Abstract and Emulate stages, where RAG-Large scored higher than both LLM-only and RAG-Small. These results complement the ANOVA findings (Table 10), confirming that retrieval augmentation has its greatest impact in stages requiring abstraction and design translation.

Stage	Comparison	Mean Difference	*p*-Value	95% CI (Lower, Upper)
Stage 1	A–B	1.022 ***	<0.001	0.402, 1.643
	A–C	–0.411	0.26	−1.032, 0.210
	B–C	–1.433 ***	<0.001	−2.054, −0.813
Stage 2	A–B	0.778 *	0.024	0.086, 1.469
	A–C	0.467	0.247	−0.225, 1.158
	B–C	−0.311	0.533	−1.002, 0.380
Stage 3	A–B	0.033	0.991	−0.578, 0.644
	A–C	−1.311 ***	<0.001	−1.922, −0.700
	B–C	−1.344 ***	<0.001	−1.956, −0.733
Stage 4	A–B	0.689 *	0.044	0.015, 1.363
	A–C	−0.844 *	0.01	−1.519, −0.170
	B–C	−1.533 ***	<0.001	−2.208, −0.859
Stage 5	A–B	−0.1	0.922	−0.720, 0.520
	A–C	−1.078 ***	<0.001	−1.698, −0.457
	B–C	−0.978 **	0.001	−1.598, −0.357
Stage 6	A–B	−0.111	0.909	−0.747, 0.525
	A–C	−1.078 ***	<0.001	−1.714, −0.442
	B–C	−0.967 **	0.001	−1.602, −0.331

Note. Asterisks denote significance (*p* < 0.05 = *, *p* < 0.01 = **, *p* < 0.001 = ***).

**Table 13:** Summary of the three-phase image generation process, highlighting distinct objectives, AI usage levels, and outputs.

Phase	Primary Inputs	Key Tools and Settings	AI Usage Level	Primary Outputs
1: Basic Image Generation	Boxfish CAD side-view silhouette; design prompts emphasizing form flow and stability	Vizcom ‘Car Shading’ palette; adjusted prompt weighting	High: LLM-RAG used to generate descriptive prompts guiding form variation	Conceptual form exploration: Multiple stylized body design sketches translating natural forms into industrial vocabulary
2: Functional Component Integration	Phase 1 outputs; manual addition of essential vehicle structures (windows, wheels)	Vizcom ‘Car Shading’ palette for configuration extension	Low: minimal reliance on LLM-RAG; emphasis on manual functional integration	Functional feasibility: Feasible vehicle body designs with integrated functional components
3: Design Detail Enhancement	Phase 2 outputs; descriptive prompts referencing biomimetic materials and finishes	Vizcom ‘Realistic Product’ and ‘Exterior’ palettes; varied ‘style influence’	Moderate: LLM-RAG used for targeted material and appearance descriptions	Visual and material refinement: High-fidelity, presentation-ready concept sketches with varied colors, proportions, and details

**Table 14:** Intraclass correlation coefficients (ICCs) for design quality ratings (N = 4). A two-way random-effects model with absolute agreement was applied. Single-measure ICC(2,1) values indicated low to moderate consistency among individual raters, while average-measure ICC(2,k) values showed strong reliability when ratings were aggregated. These results suggest that although design quality judgments varied across individual experts, consensus scores provided a stable and reliable basis for evaluation.

Measure Type	ICC	95% CI (Lower, Upper)	*F*	df1	df2	*p*
Single measures (ICC[2,1])	0.440	0.038, 0.865	4.143	5	15	0.015
Average measures (ICC[2,k])	0.759	0.137, 0.962	4.143	5	15	0.015

Note. ICCs were calculated using a two-way random-effects model with absolute agreement definition (ICC[2,k]). “Single measures” indicates the reliability of one rater, whereas “Average measures” indicates the reliability of the mean ratings across raters. Statistical significance evaluated at *p* = 0.05.

**Table 15:** Descriptive statistics for the questionnaire items (N = 4). Results include means and standard deviations across items evaluating participants’ perceptions of AI-assisted biomimicry design. The small sample size reflects the expert group involved, and the statistics provide preliminary but valuable insights into usability, perceived creativity support, and system reliability.

Item	N	Mean	SD	95% CI (Lower, Upper)
1. Adherence to the bionic concept	4	6.500	0.580	5.580, 7.420
2. Discernibility of the inspiration	4	6.000	0.820	4.700, 7.300
3. Novelty of the design	4	5.500	0.580	4.580, 6.420
4. Stylistic alignment with intended imagery	4	6.250	0.500	5.450, 7.050
5. Innovation of structures or functions	4	5.250	0.960	3.730, 6.770
6. Potential to challenge conventional vehicle concepts	4	5.500	1.290	3.510, 7.490

## Data Availability

Data available on request from the authors.

## Abbreviations

The following abbreviations are used in this manuscript:

AI	Artificial Intelligence
BDS	Biomimicry Design Spiral
CMF	Color–Material–Finish
DDM	Double Diamond model
FAISS	Facebook AI Similarity Search
GPU	Graphics Processing Unit
ICC	Intraclass Correlation Coefficient
LLMs	Large Language Models
RAG	Retrieval-Augmented Generation
RAM	Random Access Memory
SME	Small and Medium-sized Enterprise
T2I	Text-to-Image
VRAM	Video Random Access Memory
